# Endocannabinoid levels in peripheral blood mononuclear cells of multiple sclerosis patients treated with dimethyl fumarate

**DOI:** 10.1038/s41598-022-21807-y

**Published:** 2022-11-24

**Authors:** Alicia Sánchez-Sanz, María Posada-Ayala, Julia Sabín-Muñoz, Ismael Fernández-Miranda, Yolanda Aladro-Benito, Roberto Álvarez-Lafuente, Ana Royuela, Ruth García-Hernández, Ofir Rodríguez-De la Fuente, Julián Romero, Antonio García-Merino, Antonio José Sánchez-López

**Affiliations:** 1 Neuroimmunology Unit, Instituto de Investigación Sanitaria Puerta de Hierro-Segovia de Arana, Madrid, Spain; 2 PhD Program in Molecular Biosciences, Doctoral School, Universidad Autónoma de Madrid, Madrid, Spain; 3 Faculty of Experimental Sciences, Universidad Francisco de Vitoria, Madrid, Spain; 4 Department of Neurology, Hospital Universitario Puerta de Hierro Majadahonda, Madrid, Spain; 5 Lymphoma Research Group, Instituto de Investigación Sanitaria Puerta de Hierro-Segovia de Arana, Madrid, Spain; 6 Department of Neurology, Hospital Universitario de Getafe, Madrid, Spain; 7 Grupo de Investigación de Factores Ambientales en Enfermedades Degenerativas, Instituto de Investigación Sanitaria del Hospital Clínico San Carlos, Madrid, Spain; 8 Red Española de Esclerosis Múltiple (REEM), Barcelona, Spain; 9 Clinical Biostatistics Unit, Instituto de Investigación Sanitaria Puerta de Hierro-Segovia de Arana, Madrid, Spain; 10 Department of Medicine, Universidad Autónoma de Madrid, Madrid, Spain; 11 Biobank, Instituto de Investigación Sanitaria Puerta de Hierro-Segovia de Arana, Madrid, Spain

**Keywords:** Immunology, Neuroscience, Diseases, Molecular medicine, Neurology

## Abstract

The endocannabinoid system (ECS), a signalling network with immunomodulatory properties, is a potential therapeutic target in multiple sclerosis (MS). Dimethyl fumarate (DMF) is an approved drug for MS whose mechanism of action has not been fully elucidated; the possibility exists that its therapeutic effects could imply the ECS. With the aim of studying if DMF can modulate the ECS, the endocannabinoids 2-arachidonoylglycerol (2-AG), anandamide (AEA), oleoylethanolamide (OEA) and palmitoylethanolamide (PEA) were determined by liquid chromatography-mass spectrometry in peripheral blood mononuclear cells from 21 healthy donors (HD) and 32 MS patients at baseline and after 12 and 24 months of DMF treatment. MS patients presented lower levels of 2-AG and PEA compared to HD. 2-AG increased at 24 months, reaching HD levels. AEA and PEA remained stable at 12 and 24 months. OEA increased at 12 months and returned to initial levels at 24 months. Patients who achieved no evidence of disease activity (NEDA3) presented the same modulation over time as EDA3 patients. PEA was modulated differentially between females and males. Our results show that the ECS is dysregulated in MS patients. The increase in 2-AG and OEA during DMF treatment suggests a possible role of DMF in ECS modulation.

## Introduction

Dimethyl fumarate (DMF) is a first-line drug approved for relapsing–remitting multiple sclerosis (RRMS)^1^, an autoimmune disease of the central nervous system (CNS) which is more prevalent in women than in men^2^. DMF exerts immunomodulatory properties by targeting the peripheral immune system and producing a shift towards an anti-inflammatory profile^3–5^. As shown on clinical and neuroimaging parameters, a significant percentage of multiple sclerosis (MS) patients do not respond to DMF^6^. Identifying biomarkers that predict treatment response to DMF is essential for selecting the appropriate treatment for each patient and preventing relapses and disability progression^7^.

Increasing evidence suggests that DMF-induced immunological changes could be related to cell metabolism, as DMF can regulate aerobic glycolysis^8^ and lipid metabolism through changes in fatty acid levels^9^. Lipid metabolism has been linked to immune cell differentiation, effector functions, and survival^10^. Furthermore, altered lipid profiles have been associated with the pathogenesis of MS^11^ and have been studied as candidate biomarkers of disease progression^12,13^.

Part of the complex cellular lipid network is the endocannabinoid system (ECS). The ECS consists of lipid metabolites (the endocannabinoids, ECBs), the cannabinoid receptors (CBRs), and all the enzymes implicated in their synthesis and degradation^14^. The ECBs are signalling lipids that show immune-modulatory properties and, therefore, have the potential of being exploited as therapeutic targets in autoimmune diseases, including MS^15^. Moreover, there is evidence of a dysregulated ECS in the cerebrospinal fluid (CSF)^16^, plasma^17^, and peripheral lymphocytes^18^ of MS patients.

Previous work from our group showed that interferon-beta treatment can down-regulate the expression of the CBRs CB1 and CB2 and the ECBs 2-arachidonylglycerol (2-AG) and anandamide (AEA) in lymphocytes from MS patients^19^. However, no studies have evaluated the modulation of the ECS in MS patients by any of the other approved disease-modifying therapies (DMTs). In addition, DMF is able to modulate the expression in microglia cells of the CBR CB2^20^, which exerts anti-inflammatory properties^21^. This led us to investigate if DMF could be exerting immunomodulatory effects in MS patients through the modulation of the ECS.

With this aim, we have used liquid chromatography-mass spectrometry (LC–MS) to determine the levels of 4 of the most studied ECBs: 2-AG, AEA, oleoylethanolamide (OEA) and palmitoylethanolamide (PEA), in peripheral blood mononuclear cells (PBMCs) of MS patients at baseline and after 12 and 24 months of DMF treatment. Patients were clinically followed for two years to correlate clinical outcomes with DMF-induced changes in the ECS that could be useful in predicting clinical response to DMF in MS patients.

## Methods

### Patients and healthy donors

Enrolment was limited to patients diagnosed with RRMS according to McDonald criteria^22^ and with an indication for treatment with DMF^1^. Exclusion criteria included having received steroid treatment in the previous month or any kind of immunosuppressant in the previous year. Patients switching from previous treatments started DMF after a median washout period of 3 months. Cannabis users or patients under therapy with cannabinoids (Sativex or others) were also excluded from the study. A total of 32 patients were recruited from the Multiple Sclerosis Unit at Hospital Universitario Puerta de Hierro Majadahonda (HUPHM) and Hospital Universitario de Getafe (HUG). All patients signed an informed consent before participating in the study, which was approved by the Puerta de Hierro Clinical Research Ethics Committee. Patients were treated with 240 mg DMF twice a day, according to the current recommendations of use for DMF^1^. Venous blood samples were collected in tubes containing lithium heparin (Greiner Bio-One) immediately before starting treatment with DMF, at 12 months after initiating therapy, and at 24 months from 11 patients, during the scheduled follow-up visits.

Twenty-one healthy donors (HD) were also included in the study. HD were defined as individuals not affected by MS or other autoimmune disorders and were matched for age and sex with the group of MS patients. Samples and data from patients and HD in this study were provided by the Biobank HUPHM/Instituto de Investigación Sanitaria Puerta de Hierro-Segovia de Arana (PT17/0015/0020 in the Spanish National Biobanks Network), they were processed following standard operating procedures with the appropriate approval of the Ethics and Scientific Committees. Supplementary Table S1 summarizes the number of samples obtained in each hospital.

### Clinical response and MRI measures

Expanded disability status scale (EDSS) and annualized relapse rate (ARR) were evaluated in all patients at baseline, 12 and 24 months after starting therapy with DMF. A 1.5 T brain magnetic resonance imaging (MRI) was performed at the same time points to measure the number of gadolinium-enhanced T1 lesions (GdE) and the number of new or enlarged T2-weighted lesions (T2w). MRI activity was defined as the appearance of new GdE and or T2w lesions. Confirmed disability progression (CDP) was defined as an increase of at least one EDSS point sustained for three months. Clinical activity was defined as at least one relapse, exempting the first six months of DMF treatment. No evidence of disease activity 3 (NEDA-3) was calculated according to published parameters (no MRI activity, no relapses, and no CDP)^23^. Patients who achieved NEDA-3 status were defined as responders at two years, and patients who did not achieve it (evidence of disease activity, EDA-3) were defined as non-responders at two years.

### PBMC isolation and LC–MS

PBMCs were isolated from peripheral blood, immediately as it was collected, by Ficoll density gradient centrifugation according to the manufacturer’s protocol and cryopreserved in liquid nitrogen until use. PBMC samples were prepared for LC–MS using acetonitrile for protein precipitation and homogenized in methanol containing 5 μl of the deuterated internal standards 2-AG-d8, 1-arachidonoylglycerol-d8 (1-AG-d8), AEA-d8, OEA-d4 and PEA-d5 (Cayman Chemical). Lipids were extracted with chloroform:H2O (2:1) containing 0.1% formic acid. The organic phase was collected and dried in SpeedVac (Thermo Scientific) at 55 ºC, and samples were reconstituted in methanol. Data were acquired using Acquity UPLC H-Class (Waters) and QTrap 4500 systems (Sciex), using an Acquity HSS T3 column (1.2 × 100 mm and 1.8 µm, Waters) at 30ºC and a mobile phase composed of formic acid 0.1% and acetonitrile. Data were analysed using Analyst software 1.6 (Sciex). For ECB quantification, two calibration curves were performed using commercial ECB standards, and peak areas were normalized using the internal standards with MultiQuant software (Sciex). The concentration of each ECB was normalized to the total amount of protein in each sample (pmol/g of protein), which was quantified using the BCA protein assay (Thermo Scientific). This normalization allowed us to prevent observed sample variability and confounder effects due to a decrease in the absolute lymphocyte count (ALC) produced by DMF treatment. As it has been described that DMF causes a reduction in ALC and even lymphopenia in a number of patients^6^, we collected ALC data from patients’ hemograms at baseline and 12 months. In our cohort, DMF produced a decrease in ALC at 12 months, but none of the patients developed severe lymphopenia (< 0.5 × 10^3^/µl) (Supplementary Fig. 1A). Correlation analyses showed no relationships between ALC and ECBs neither at baseline nor at 12 months (Supplementary Figs. 1B–E and 2).

### Statistical and bioinformatics analysis

Statistical analyses were performed using Graphpad Prism 8 and STATA 13 software. Patients were stratified according to clinical response, sex and treatment status (naïve or not). For individual analyses, we assessed statistical significance using the Mann–Whitney test for comparisons between HD and patients at baseline and between subgroups (Responder vs. Non-responder, Women vs. Men, or Naïve vs. Previously treated). The Wilcoxon signed-rank test was used for paired samples between the different time points and baseline. Statistical significance was established at p < 0.05. Bioinformatic analyses were performed using R 3.6.1 (https://www.R-project.org, R Foundation for Statistical Computing, Vienna, Austria). Principal component analysis (PCA) and hierarchical clustering using the Euclidean distance were performed using the concentrations of 2-AG, AEA, OEA and PEA at baseline as an exploratory analysis between HD and MS patients.

### Ethics approval and consent to participate

The study was conducted according to the guidelines of the Declaration of Helsinki, and approved by the Ethics Committee of HUPHM. Informed consent was obtained from all subjects involved in the study.

## Results

### Clinical and MRI data

Table 1 shows the demographic characteristics and clinical data at baseline from MS patients and HD, and the three subgroups of patients. The mean age of our cohort of patients was ≈ 38 years, with a disease duration of ≈ 6 years. Seventy-five percent (n = 24) were female. About 50% (n = 17) were naïve, while patients with previous treatments had received interferon beta (n = 12), glatiramer acetate (n = 2) or natalizumab (n = 1). In the subgroup of previously treated patients, ≈ 90% (n = 13) had received only one previous DMT, and 60% (n = 9) had switched to DMF due to tolerability and 40% (n = 6) due to lack of efficacy. The three subgroups of patients presented similar demographic and clinical characteristics at baseline, except for naïve MS patients who, as expected, presented a significantly shorter disease duration (p = 0.0002) and a higher ARR (p = 0.0006) compared to previously treated patients. Supplementary Table S2 summarizes the clinical and MRI measures obtained at 1 and 2 years of DMF treatment. At the end of the study, DMF reduced the mean ARR (p < 0.0001), as well as the number of GdE and T2w lesions (p = 0.0303 and p = 0.0083, respectively). The percentage of NEDA-3 patients at 2 years was 68.8% (n = 22). None of the patients discontinued DMF treatment during the follow-up. Similar results were found in the subgroups of patients. However, it should be noted that the subgroup of men did not significantly reduce the mean ARR or the number of GdE or T2w lesions neither at one nor at two years, which could be indicating a suboptimal response to DMF in male patients.

**Table 1:** Demographic and clinical characteristics of multiple sclersosis (MS) patients at baseline and healthy donors (HD).

	Groups					
	MS patients (n = 32)	HD (n = 21)				
Age (years)^a^	37.56 ± 8.59	33.67 ± 9.44				
Sex (% of female)^b^	75%	80, 95%				
Disease duration (years)^a,c^	5.64 ± 6.34	–				
Time since DMT onset (years)^a^	2.92 ± 4.87	–				
Number of previous DMTs^a^	0.63 ± 0.98	–				
Immediately previous treatment	Naïve: 53.13%	–				
	Interferon: 37.50%	–				
	Glatiramer acetate: 6.25%	–				
	Natalizumab: 3.13%	–				
Reason for treatment change	Naïve: 53.13%	–				
	Lack of efficacy: 18.75%	–				
	Tolerability: 28.13%	–				
Basal ARR^a,d^	0.81 ± 0.81	–				
Basal EDSS^a^	1.30 ± 1.31	–				
Number of GdE lesions^a^	0.53 ± 0.92	–				
Number of new T2-Weighted lesions^a,e^	1.27 ± 0.59	–				

	MS Subgroups					
	Responder (n = 22)	Non-responder (n = 10)	Naïve (n = 17)	Previously treated (n = 15)	Women (n = 24)	Men (n = 8)
Age (years)^a^	39.05 ± 8.17	34.30 ± 9.02	35.12 ± 8.57	40.33 ± 8.01	37.88 ± 8.39	36.63 ± 9.71
Sex (% of female)^b^	81.82%	60%	76.47%	73.33%	–	–
Disease duration (years)^a,c^	6.11 ± 6.46	4.60 ± 6.29	2.78 ± 3.46***	8.87 ± 6.31***	5.61 ± 4.77	5.73 ± 6.18
Time since DMT onset (years)^a^	2.95 ± 4.71	2.85 ± 5.47	–	6.23 ± 5.52	2.76 ± 4.68	3.41 ± 5.72
Number of previous DMTs^a^	0.50 ± 0.51	0.90 ± 1.60	–	1.33 ± 1.05	0.67 ± 1.09	0.50 ± 0.53
Immediately previous treatment	Naïve: 50%	Naive: 60%	–	Interferon: 80%	Naïve: 54.17%	Naïve: 50%
	Interferon: 40.41%	Interferon: 30%	–	Glatiramer acetate: 13.33%	Interferon: 33.33%	Interferon: 50%
	Glatiramer acetate: 9.09%	Natalizumab: 10%	–	Natalizumab: 6.67%	Natalizumab: 4.17%	
			–		Glatiramer acetate: 8.33%	
Reason for treatment change	Naïve: 50%	Naïve: 60%	–		Naïve: 54.17%	Naïve: 50%
	Lack of efficacy: 13.64%	Lack of efficacy: 30%	–	Lack of efficacy: 40%	Lack of efficacy: 16.67%	Lack of efficacy: 25%
	Tolerability: 36.36%	Tolerability: 10%	–	Tolerability: 60%	Tolerability: 29.17%	Tolerability: 25%
Basal ARR^a,d^	0.68 ± 0.65	1.11 ± 1.07	1.19 ± 0.94***	0.38 ± 0.26***	0.72 ± 0.64	1.10 ± 1.20
Basal EDSS^a^	0.95 ± 0.90	2.05 ± 1.77	1.06 ± 1.06	1.57 ± 1.55	1.25 ± 1.42	1.44 ± 0.98
Number of GdE lesions^a^	0.45 ± 0.91	0.70 ± 0.95	0.82 ± 1.13	0.20 ± 0.41	0.38 ± 0.71	1.00 ± 1.31
Number of new T2-Weighted lesions^a,e^	1.18 ± 0.40	1.50 ± 1.00	–	1.27 ± 0.59	1.36 ± 0.50	1.00 ± 0.82

***p < 0.001.

^a^Values are the mean ± standard deviation (SD) of each group. The Mann–Whitney test was used to compare differences between MS and HD and between subgroups (Responder vs Non-responder, Naïve vs Previously treated or Women vs Men).

^b^Chi-Square test was used to compare two proportions.

^c^Time since the first symptoms of MS.

^d^For Basal ARR, only the previous year was considered.

^e^Data not available for naïve MS patients, as most of them had a single magnetic resonance imaging (MRI).

### Endocannabinoid levels are dysregulated in MS patients compared to HD

**Figure 1:**
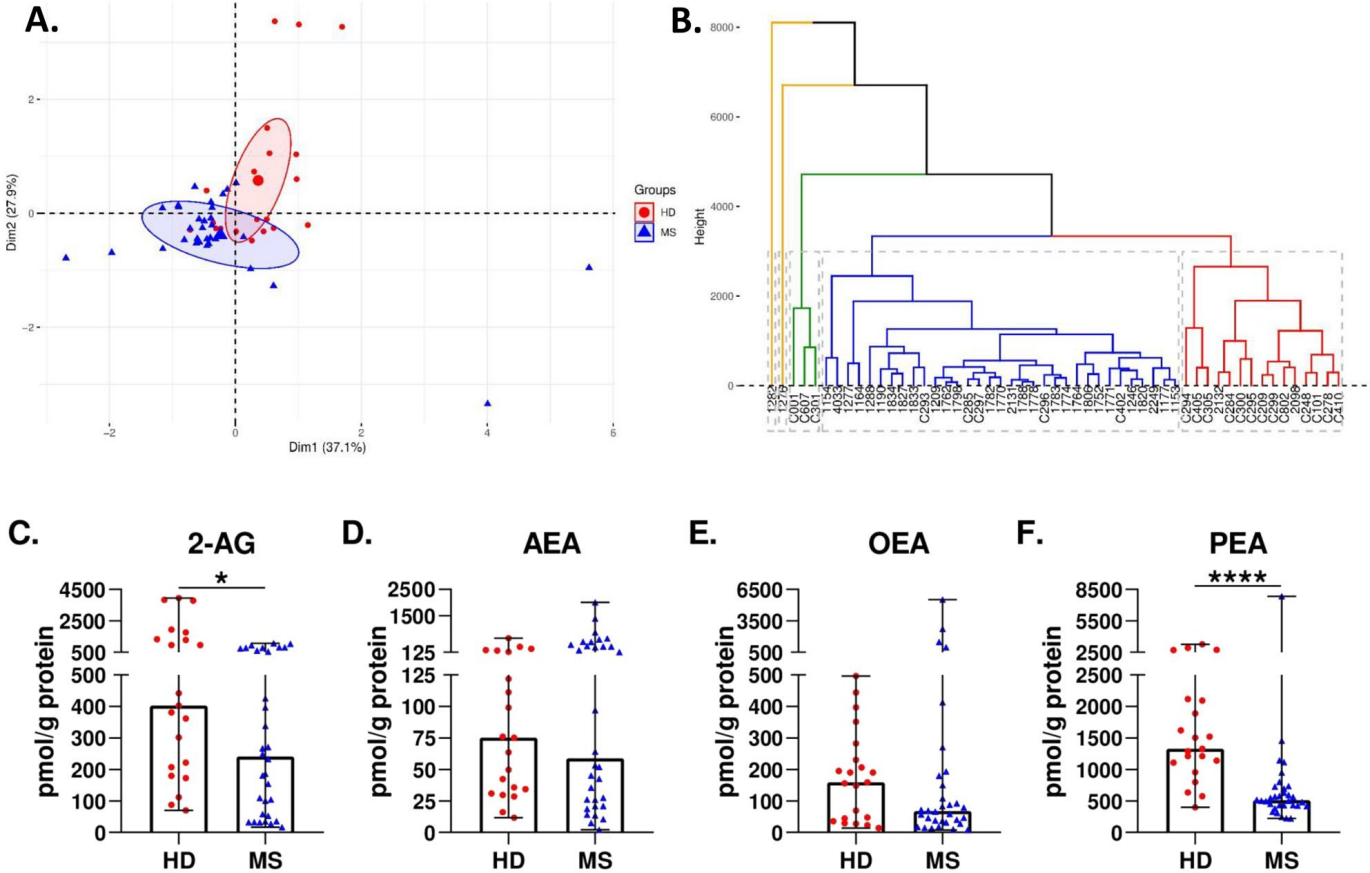
Endocannabinoid levels are dysregulated in MS patients compared to HD. (**A**) Principal component analysis of the endocannabinoid profile in HD and MS patients at baseline. HD (red) locate in a differentiated region of space compared to MS patients at baseline (blue) (**B**) Hierarchical clustering of HD and MS patients at baseline imputed from the four endocannabinoids (2-AG, AEA, OEA and PEA). HD are indicated with a C and their corresponding sample number . (**C**–**F**) Individual levels (in pmol/g of protein) of each endocannabinoid in HD and MS patients at baseline. MS patients presented lower levels of 2-AG and PEA. The Mann–Whitney test was used to compare differences between groups. Data are expressed as median with range. *p < 0.05; ****p < 0.0001; ns, not significant.

PCA shows the endocannabinoid profile of HD and MS patients at baseline (Fig. 1A). Most of the samples were similar within the group of HD or MS patients, and each group was located in a differentiated region of space, indicating that both groups present discernible endocannabinoid profiles. OEA and PEA were the ECBs that contributed more to the PCA model (Supplementary Fig. S3). Hierarchical clustering supports the same results as the PCA, as most HD formed a differentiated cluster and MS patients were distributed in another separated cluster (Fig. 1B). Furthermore, 28 out of 32 MS patients were grouped inside the same cluster, indicating high homogeneity between patients. Individual analysis of each endocannabinoid shows that 2-AG and PEA were significantly lower in patients at baseline compared to HD (p = 0.0134 and p = 0.0000, respectively) (Fig. 1C and F). Moreover, the median values of both ECBs were half the levels of HD. Although OEA levels were also half those of HD (Fig. 1E), they did not reach statistical significance (p = 0.1967). The levels of AEA (Fig. 1D) were similar between patients at baseline and HD (p = 0.7990). We found no statistical differences at baseline within the different subgroups of MS patients for any of the four ECBs (Supplementary Fig. S4).

### Characterization of 2-AG levels during DMF treatment

**Figure 2:**
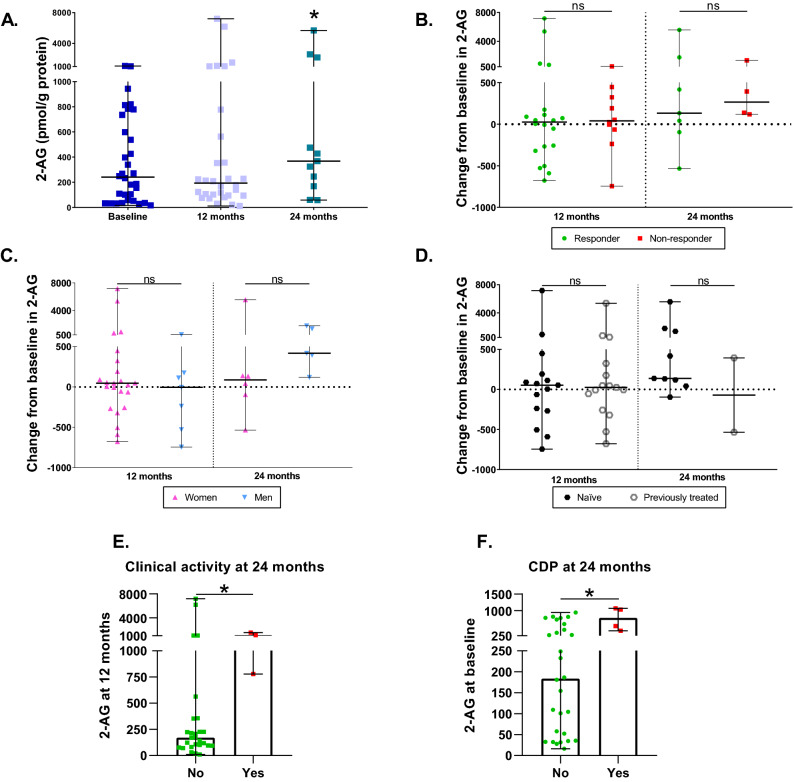
Characterization of 2-AG levels during DMF treatment. (**A**) 2-AG levels in MS patients at baseline and after 12 and 24 months of DMF treatment. (**B**) Changes in 2-AG levels at 12 and 24 months compared to baseline between responders and non-responders to DMF. (**C**) Changes in 2-AG levels at 12 and 24 months compared to baseline between women and men (**D**) Changes in 2-AG levels at 12 and 24 months compared to baseline between naïve and previously treated patients (**E**) 2-AG at 12 months in patients with (yes) or without (no) clinical activity at 24 months. (**F**) 2-AG at baseline in patients with (yes) or without (no) confirmed disease progression (CDP) at 24 months. The Wilcoxon test was used to compare differences between the different time points and baseline, and the Mann–Whitney test was used to compare differences between subgroups. Data are expressed as median with range in pmol/g of protein. *p < 0.05; ns, not significant.

During DMF treatment, 2-AG was similar to baseline at 12 months (p = 0.5706) but experienced an increase at 24 months (p = 0.0409), reaching similar levels as those of HD (Fig. 2A). The change from baseline in 2-AG did not differ between responders and non-responders at 12 and 24 months (p = 0.6921 and p = 0.7055, respectively) (Fig. 2B). Likewise, there were no differences when comparing women and men at 12 or 24 months (p = 0.5399 and p = 0.1441, respectively) (Fig. 2C). The subgroup of naïve versus previously treated patients did not present any difference at 12 or 24 months either (p = 0.9007 and p = 0.3458, respectively) (Fig. 2D). These results indicate that the changes in 2-AG during DMF treatment are similar in the three subgroups of patients. At two years, patients with CDP presented higher levels of 2-AG at baseline (p = 0.0194) (Fig. 2E); patients with clinical activity at two years also showed higher levels of 2-AG at 12 months (p = 0.0247) (Fig. 2F).

### Characterization of AEA levels during DMF treatment

**Figure 3:**
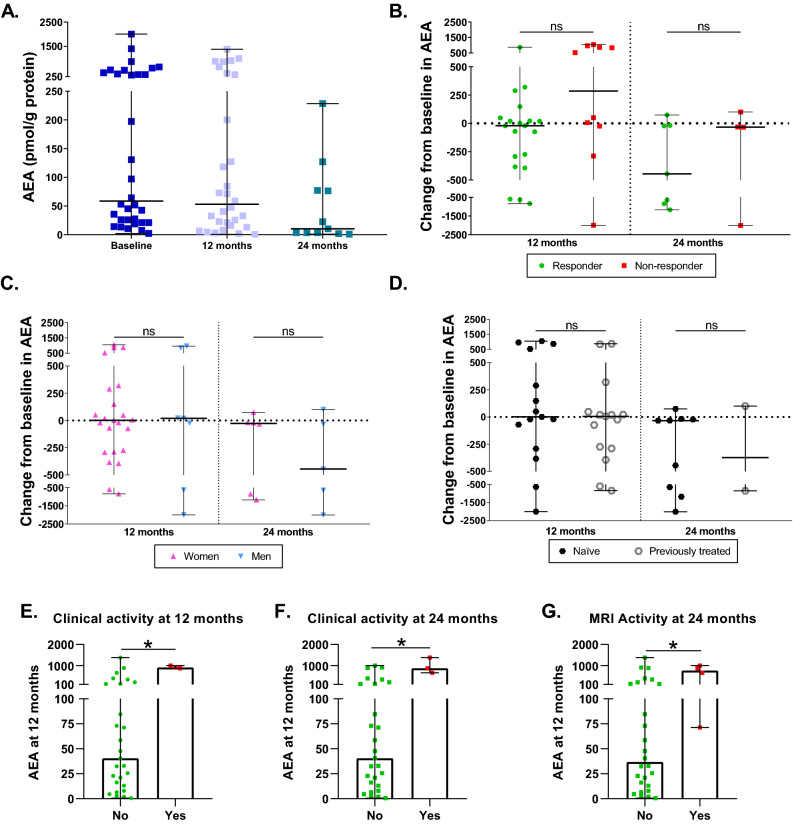
Characterization of AEA levels during DMF treatment. (**A**) AEA levels in MS patients at baseline and after 12 and 24 months of DMF treatment. (**B**) Changes in AEA levels at 12 and 24 months compared to baseline between responders and non-responders to DMF. (**C**) Changes in AEA levels at 12 and 24 months compared to baseline between women and men. (**D**) Changes in AEA levels at 12 and 24 months compared to baseline between naïve and previously treated patients. (**E**) AEA at 12 months in patients with (yes) or without (no) clinical activity at 12 months. (**F**) AEA at 12 months in patients with (yes) or without (no) clinical activity at 24 months. (**G**) AEA at 12 months in patients with (yes) or without (no) MRI activity at 24 months. The Wilcoxon test was used to compare differences between the different time points and baseline, and the Mann–Whitney test was used to compared differences between subgroups. Data are expressed as median with range in pmol/g of protein. *p < 0.05; ns, not significant.

At 12 months, AEA median values were similar to baseline (p = 0.9263), while at 24 months, AEA experienced a decrease compared to baseline almost significant (p = 0.0505) (Fig. 3A). There were no significant differences between 12 or 24 months and baseline between responders and non-responders (p = 0.0784 and p = 0.8501, respectively) (Fig. 3B). Women and men did not present any difference between baseline AEA and AEA at 12 or 24 months (p = 0.9804 and p = 0.5839, respectively) (Fig. 3C). Similarly, naïve and patients with previous treatments did not present any difference in AEA change at 12 or 24 months (p = 0.3184 and p = 0.6373) (Fig. 3D). Higher AEA levels at 12 months were found in patients with clinical activity at 1 (p = 0.0116) (Fig. 3E) and two years (p = 0.0170) (Fig. 3F) and with MRI activity (p = 0.0327) at two years (Fig. 3G).

### Characterization of OEA levels during DMF treatment

**Figure 4:**
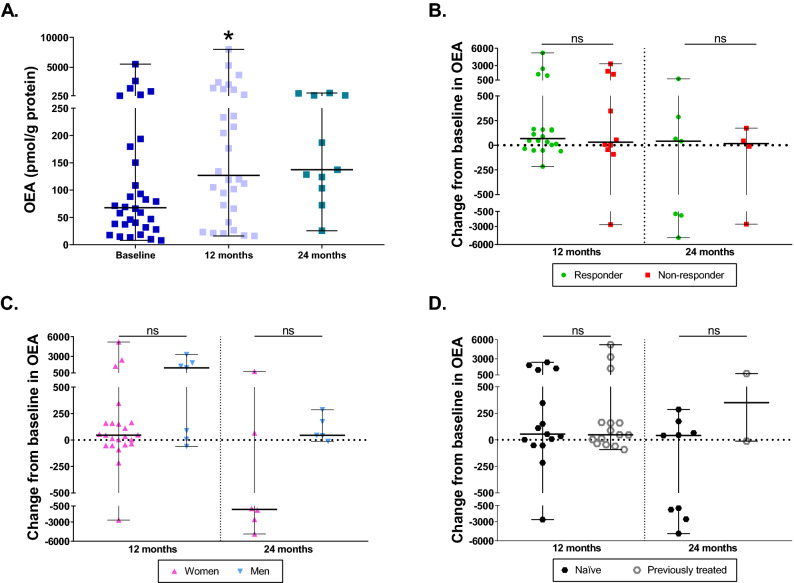
Characterization of OEA levels during DMF treatment. (**A**) OEA levels in MS patients at baseline and after 12 and 24 months of DMF treatment. (**B**) Changes in OEA levels at 12 and 24 months compared to baseline between responders and non-responders to DMF. (**C**) Changes in OEA levels at 12 and 24 months compared to baseline between women and men. (**D**) Changes in OEA levels at 12 and 24 months compared to baseline between naïve and previously treated patients. The Wilcoxon test was used to compare differences between the different time points and baseline, and the Mann–Whitney test was used to compared differences between subgroups. Data are expressed as median with range. *p < 0.05; ns, not significant.

At 12 months, OEA levels raised above baseline (p = 0.0125) (Fig. 4A), reaching a median more similar to the one of HD. At 24 months, the median of OEA remains equal to 12 months; however, no statistical differences were achieved compared to baseline (p = 0.5937) (Fig. 4A). No differences were found between responders and non-responders in the change of OEA at 12 or 24 months compared to baseline (p = 0.8603 and p = 0.8501, respectively) (Fig. 4B), as well as between women and men (p = 0.1623 at 12 months and p = 0.8312 at 24 months) (Fig. 4C) and between naïve or patients who had received previous treatments (p = 0.9337 at 12 months and p = 0.3458 at 24 months) (Fig. 4D). No association was found between OEA and any of the individual clinical or MRI variables.

### Characterization of PEA levels during DMF treatment

**Figure 5:**
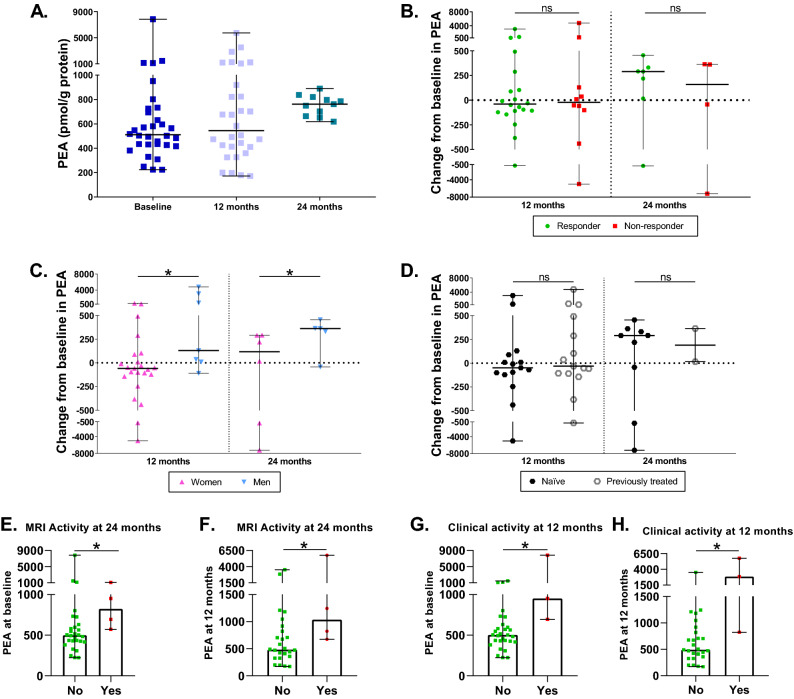
Characterization of PEA levels during DMF treatment. (**A**) PEA levels in MS patients at baseline and after 12 and 24 months of DMF treatment. (**B**) Changes in PEA levels at 12 and 24 months compared to baseline between responders and non-responders to DMF. (**C**) Changes in PEA levels at 12 and 24 months compared to baseline between women and men. (**D**) Changes in PEA levels at 12 and 24 months compared to baseline between naïve and previously treated patients. (**E**) PEA at baseline in patients with (yes) or without (no) MRI activity at 24 months. (**F**) PEA at 12 months in patients with (yes) or without (no) MRI activity at 24 months. (**G**) PEA at baseline in patients with (yes) or without (no) clinical activity at 12 months (**H**) PEA at 12 months in patients with (yes) or without (no) clinical activity at 12 months. The Wilcoxon test was used to compare differences between the different time points and baseline, and the Mann–Whitney test was used to compared differences between subgroups. Data are expressed as median with range. *p < 0.05; ns, not significant.

At 12 and 24 months, PEA had baseline levels (p = 0.8612 and p = 0.3739 respectively) (Fig. 5A). Responder and non-responder patients did not differ in PEA levels neither at 12 nor at 24 months (p = 0.8259 and p = 0.8501, respectively) (Fig. 5B). Interestingly, when classifying patients by sex, we could observe that PEA varied differentially within this subgroup (Fig. 5C). Men presented an increase in PEA at 12 months (p = 0.0174) and 24 months of DMF treatment (p = 0.0446), absent in women. Naïve and patients with previous treatments did not show any difference at 12 (p = 0.8030) or 24 months (p = 0.6373) (Fig. 5D). At two years, patients with MRI activity presented higher levels at baseline and 12 months of PEA (p = 0.0461 and p = 0.0380, respectively) (Fig. 5E, F). Patients with clinical activity at one year also presented higher PEA levels at baseline and 12 months (p = 0.0257 and p = 0.0205, respectively) (Fig. 5G, H).

## Discussion

Our results confirm that MS patients present a dysregulated ECS, as shown by the lower levels of 2-AG and PEA in patients at baseline compared to HD. The presence of an altered ECS tone highlights the potential of targeting the ECS in MS therapy. In this regard, and to the best of our knowledge, this is the first study evaluating the ECS regulation by DMF in MS patients. We have seen that, during DMF treatment, at least two ECBs (2-AG and OEA) are modulated and that this modulation fluctuates depending on the endocannabinoid and treatment time. Furthermore, the endocannabinoid tone during treatment does not seem to be associated with clinical response to DMF.

Although both positive and negative effects have been attributed to ECBs, increasing evidence supports a protective role of the ECS in neuroinflammatory conditions^24^. 2-AG modulates the immune responses through CB2 by inhibiting cell recruitment and enhancing the production of anti-inflammatory cytokines^25–27^. Furthermore, the inhibition of 2-AG hydrolysis can protect the blood–brain barrier (BBB) permeability in inflammatory conditions^28^. PEA has also been linked to anti-inflammatory properties by inhibiting the activation of mast cells^29^ and the regulation of CB2 expression in macrophages and microglia^30^. In rats’ acute inflammation, exogenous administration of PEA showed therapeutic effects by inhibiting the production of nitric oxide (NO) and cyclo-oxygenase activity (COX) at the site of inflammation^31^. In the mouse model of chronic relapsing experimental autoimmune encephalomyelitis (EAE), PEA increased in the damaged nerve areas^32^, and it might have a relevant role in counteracting inflammation and maintaining homeostasis^33^. The presence of lower levels of 2-AG and PEA in our cohort of patients could be indicating that the endogenous production of these ECBs is deficient and hence, insufficient to control inflammation in MS.

Moreover, the presence of an altered endocannabinoid tone in MS patients compared to HD also highlights the potential of using ECBs as biomarkers of disease. Lipidomic studies have already proven to help identify MS patients^34^. In the mentioned study, serum concentrations of 43 different lipids were measured in HD and MS, and the eight lipids that could best predict MS were selected to create a classifier with an accuracy of 95%. Interestingly, among the selected lipids were PEA and OEA, which were also the two ECBs that contributed more to our PCA, reinforcing a functional relevance for ECBs in MS. In our study, we have seen that using only our four ECBs, MS patients could also be distinguished from HD. Furthermore, as the results we have found in PBMCs are similar to those found in serum^34^, this could be indicating that measuring ECBs in serum could be an indirect estimation of ECBs production by PBMCs.

During DMF treatment, the levels of PEA were not restored and remained low during the 24 months of follow-up. As for 2-AG levels, they experienced a slight increase at 24 months, although it is uncertain whether this gain is directly produced by DMF, as most of the therapeutic effects of the drug are achieved during the first months of therapy and because of the smaller sample size at 24 months. New therapies based on increasing the ECB tone, such as administering exogenous ECBs or hydrolysis inhibitors have demonstrated beneficial anti-inflammatory effects in EAE mice treated with PEA^35^ and in murine models of EAE^36^, Alzheimer’s disease^37^, rheumatoid arthritis^38^ and airway inflammation^39^ treated with inhibitors of the degradative ECB enzymes monoacylglycerol lipase (MAGL), fatty acid amide hydrolase (FAAH) or α/β-hydrolase domain containing 6 (ABHD6).

The ECB that increased more significantly during DMF treatment was OEA. Although OEA was not statistically dysregulated compared to HD, it contributed considerably to the PCA, indicating that it might be differentially expressed in MS patients. We think that OEA modulation could be of particular relevance as this ECB has been related to the Nrf2/Heme oxygenase-1 (HO-1) pathway^40^, one of the proposed mechanisms of action of DMF. Nrf2 is a transcription factor involved in redox homeostasis through the expression of cytoprotective and antioxidant genes and has been studied as a therapeutic target in neuroinflammation^41^. In a murine model of liver injury, OEA therapy inhibited the number of activated macrophages, the production of pro-inflammatory cytokines and oxidative stress via up-regulation of Nrf2^40^. Regarding its effects on the immune system, OEA is able of inhibiting both CD4 and CD8 T lymphocytes by reducing the production of TNF-α, IFN-γ, and IL-17^42^. Moreover, it has been found that oleic acid, precursor of OEA, is able to restore suppressive defects in regulatory T cells from patients with MS^43^. Taking all this into account, performing in vitro studies evaluating the molecular pathways through which DMF interacts to increase OEA levels, could shed light on the mechanism of action of DMF.

Furthermore, OEA has been extensively studied in the context of the gastro-intestinal tract. OEA treatment can alter the gut microbiota composition, modulating immune responses in the Peyer’s Patches^44^. DMF is an oral drug that is metabolized in the intestine^45^. Accordingly, it could mediate relevant effects in this organ contributing to the immune modulatory effects achieved by this drug. The number of studies evaluating the effects of DMF on the gut microbiota of MS patients is still scarce, but they are suggestive of DMF-induced changes in the gut microbial composition ^46,47^. The increase we have seen in OEA in PBMCs during DMF treatment highlights the need of further studies evaluating if some of the therapeutic effects of DMF could be linked to an increase in OEA in the gut, that could be affecting the gut microbiota.

AEA levels have been reported to be both higher^18,19^ and lower^16,34^ in MS patients compared to HD. In our cohort of patients, AEA levels were the same as in HD, which could be due to the dispersion of the data and to the sample size of our cohort. In addition, DMF does not seem to affect AEA levels as they remained unaltered during the 24 months of follow-up.

Regarding the clinical response, we have not found any differences between responder and non-responder patients to DMF in any of the four ECBs when using the NEDA-3 criteria to classify patients. This finding could be due to the small number of EDA3 patients and to the clinical heterogeneity between MS patients, a hallmark of the disease, that could hinder the identification of response biomarkers. However, we have seen several associations in ECB levels with individual clinical parameters such as clinical and MRI activity. Previous studies have demonstrated increased ECB levels associated with GdE lesions^16^ and the acute phase of experimental autoimmune encephalomyelitis^18^. This could be indicating that changes in ECB levels could be related to specific processes of the disease, such as increased BBB permeability and relapses, highlighting the importance of studying ECB levels at the appropriate time point. The possible temporal relationship between clinical worsening and ECB levels was unlikely, as all samples in the study were collected during the pre-specified follow-up visits. In cases of unscheduled visits due to relapses or clinical deterioration, no samples were obtained.

As for the subgroup of naïve or previously treated patients, we wanted to see if receiving any previous treatment could have influenced the endocannabinoid tone in MS patients. Most of the previously treated patients had received only one previous DMT and most of them were switching from interferon beta, which is able to decrease 2-AG and AEA^19^. However, no baseline differences were found in any ECB, indicating that the modulation produced by interferon is lost in the wash-out period that patients undergo between treatments. The modulation produced duringDMF treatment was not affected either by previous treatments.

Previous studies have reported differences in ECB levels depending on MS subtype^16,17^. Patients with secondary progressive MS (SPMS) presented a trend towards lower ECB values in CSF compared to RRMS^16^. In plasma, differences in ECB levels were also found between RRMS, primary progressive MS (PPMS) and SPMS^17^. These results suggest that differences in the ECS are present depending on the disease stage. In this regard, PPMS and SPMS are characterized by a neurodegenerative disease state with sustained disease worsening without relapses, in contrast with RRMS. In our study, the lack of differences in ECB levels between naïve and previouly treated patients could be explained by the fact the all of them had RRMS, a disease stage where inflammation is the predominant trait.

Finally, it is interesting to notice the differences found in PEA between women and men, which presented an increase in PEA during DMF treatment that was absent in women. In MS, women present a higher prevalence of RRMS, with a 3:1 preponderance^48^. Women are also diagnosed earlier and have a different inflammatory pattern with more relapses and MRI activity. In contrast, men present a worse disease course with faster progression and less inflammatory activity but greater atrophy of the grey matter ^48^. We find the results in PEA intriguing, as this ECB was more elevated in male patients, which presented a worse response to DMF, and because higher PEA was also associated with MRI and clinical activity. This could be due to a compensatory mechanism in patients with worse inflammatory patterns in which PEA would increase to counteract inflammation.

In addition, sexual dimorphisms in the ECS have already been reported, such as a differential expression of the receptor CB1 in the CNS and a differential sensitivity to cannabinoid compounds in humans^49^. Differences in 2-AG and AEA concentrations between males and females, as well as between female estrous cycle stages have also been reported in Sprague Dawley rats ^50^. However, the number of studies evaluating sex differences in the levels of ECBs is still scarce and, to the best of our knowledge, there are no studies evaluating these differences in MS. Deepening our understanding of the relationship between the ECS and sexual dimorphisms could help us understand the mechanisms underlying the sex differences found in MS.

## Conclusions

The ECS is dysregulated in peripheral lymphocytes from MS patients, as shown by the lower levels of 2-AG and PEA compared to HD. These data contribute- to the mounting evidence that points to the involvement of the ECS in the regulation of the immune system in MS and highlights the potential of the ECS as a therapeutic target. The increase in 2 endocannabinoids (2-AG and OEA) during DMF treatment suggests a possible role of DMF in the modulation of the ECS. This modulation fluctuates depending on the endocannabinoid and treatment time, and it seems not to be associated with clinical response to DMF. Further studies evaluating the direct effect of DMF on the ECS could shed light on the immunomodulatory mechanism of action of DMF.

## Supplementary Information

Supplementary Information.

## Data Availability

The datasets used and/or analysed during the current study are available from the corresponding author on reasonable request.

## Abbreviations

DMF: Dimethyl fumarate
RRMS: Relapsing–remitting multiple sclerosis
CNS: Central nervous system
MS: Multiple sclerosis
Nrf2: Nuclear factor E2-related factor 2
HCAR2: Hydroxycarboxylic acid receptor 2
ECS: Endocannabinoid system
ECB: Endocannabinoid
CBR: Cannabinoid receptor
CSF: Cerebrospinal fluid
CB1: Cannabinoid receptor 1
CB2: Cannabinoid receptor 2
2-AG: 2-Arachidonylglycerol
AEA: Anandamide
DMT: Disease modifying therapy
LC–MS: Liquid chromatography–mass spectrometry
OEA: Oleoylethanolamide
PEA: Palmitoylethanolamide
PBMCs: Peripheral blood mononuclear cells
HUPHM: Hospital Universitario Puerta de Hierro Majadahonda
HUG: Hospital Universitario de Getafe
HD: Healthy donor
EDSS: Expanded disability status scale
ARR: Annualized relapse rate
MRI: Magnetic resonance imaging
GdE: Gadolinium-enhanced T1 lesions
T2w: T2-weighted lesions
CDP: Confirmed disability progression
NEDA-3: No evidence of disease activity 3
EDA-3: Evidence of disease activity 3
1-AG: 1-Arachidonoylglycerol
GEE: Generalized estimating equations
ALC: Absolute lymphocyte count
PCA: Principal component analysis
BBB: Blood–brain barrier
NO: Nitric oxide
COX: Cyclo-oxygenase
EAE: Experimental autoimmune encephalomyelitis
MAGL: Monoacylglycerol lipase
FAAH: Fatty acid amide hydrolase
ABHD6: α/β-Hydrolase domain containing 6
HO-1: Heme-oxygenase 1
