# Supplementary material for: Endocannabinoid levels in peripheral blood mononuclear cells of multiple sclerosis patients treated with dimethyl fumarate
Source: Sci Rep. 2022 Nov 24;12:20300. doi: 10.1038/s41598-022-21807-y (PMC9700785; doi:10.1038/s41598-022-21807-y)
Supplement: Supplementary file 1 — Supplementary Information. [file 41598_2022_21807_MOESM1_ESM.pdf]

SUPPLEMENTARY TABLE S1. Number of samples obtained in each hospital

	HD (n)	MS patients (n)		
**Hospital**	**Single Time Point**	**Baseline**	**12 months**	**24 months**
**HUPHM**	21	20	20	0
**HUG**	0	12	12	11
**Total**	21	32	32	11

Samples from healthy donors (HD) were obtained at a single time point. Samples from multiple sclerosis (MS) patients were obtained immediately before starting dimethyl fumarate (DMF) treatment (baseline) and after 12 and 24 months of therapy with DMF.

HUPHM, Hospital Universitario Puerta de Hierro Majadahonda; HUG, Hospital Universitario de Getafe

SUPPLEMENTARY TABLE S2. Clinical and MRI outcomes

	Time periods			Statistical significance		
	**Baseline**	**1 year**	**2 years**	**Baseline vs 1 year**	**1 year vs 2 years**	**Baseline vs 2 years**
**ARR†,‡**						
**All**	0.81 ± 0.81	0.50 ± 0.88	0.30 ± 0.51	**0.0014**	0.0605	**<0.0001**
**Responder**	0.68 ± 0.65	0.36 ± 0.66	0.20 ± 0.40	**0.0013**	**0.0313**	**<0.0001**
**Non-responder**	1.11 ± 1.07	0.80 ± 1.23	0.50 ± 0.67	0.2500	0.6250	0.2402
**Naïve**	1.19 ± 0.94	0.82 ± 1.07	0.47 ± 0.60	**0.0127**	0.0938	**0.0016**
**Previously treated**	0.38 ± 0.26	0.13 ± 0.35	0.10 ± 0.28	**0.0494**	>0.9999	**0.0085**
**Women**	0.72 ± 0.64	0.29 ± 0.62	0.27 ± 0.55	**0.0001**	0.5625	**0.0003**
**Men**	1.10 ± 1.20	1.13 ± 1.25	0.38 ± 0.35	>0.9999	0.0625	0.1484
**EDSS†**						
**All**	1.30 ± 1.31	1.14 ± 1.25	1.11 ± 1.35	0.0742	0.6523	0.0829
**Responder**	0.95 ± 0.90	0.82 ± 0.84	0.75 ± 0.94	0.0625	0.4375	0.0781
**Non-responder**	2.05 ± 1.77	1.85 ± 1.70	1.90 ± 1.79	0.5000	>0.9999	0.6719
**Naïve**	1.06 ± 1.06	0.85 ± 0.86	0.88 ± 0.93	0.0625	>0.9999	0.3438
**Previously treated**	1.57 ± 1.55	1.47 ± 1.54	1.37 ± 1.71	0.6250	0.4375	0.1719
**Women**	1.25 ± 1.42	1.06 ± 1.35	1.13 ± 1.48	**0.0156**	0.5938	0.3389
**Men**	1.44 ± 0.98	1.38 ± 0.92	1.06 ± 0.94	>0.9999	0.2500	0.2500
**Number of GdE lesions†**						
**All**	0.53 ± 0.92	0.00 ± 0.00	0.13 ± 0.55	**0.0010**	0.5000	**0.0303**
**Responder**	0.45 ± 0.91	0.00 ± 0.00	0.00 ± 0.00	**0.0313**	>0.9999	**0.0313**
**Non-responder**	0.70 ± 0.95	0.00 ± 0.00	0.40 ± 0.97	0.0625	0.5000	0.5625
**Naïve**	0.82 ± 1.13	0.00 ± 0.00	0.06 ± 0.93	**0.0078**	>0.9999	**0.0078**
**Previously treated**	0.20 ± 0.41	0.00 ± 0.00	0.20 ± 0.77	0.2500	>0.9999	>0.9999
**Women**	0.38 ± 0.71	0.00 ± 0.00	0.00 ± 0.00	**0.0156**	>0.9999	**0.0156**
**Men**	1.00 ± 1.31	0.00 ± 0.00	0.50 ± 1.07	0.1250	0.5000	0.5000
**Number of new T2-weighted lesions†,§**						
**All**	1.27 ± 0.59	0.44 ± 1.05	0.28 ± 0.89	**0.0002**	0.3438	**0.0083**
**Responder**	1.18 ± 0.40	0.25 ± 0.85	0.04 ± 0.20	**0.0010**	0.2500	**0.0010**
**Non-responder**	1.50 ± 1.00	1.00 ± 1.41	1.00 ± 1.60	0.5000	>0.9999	>0.9999
**Naïve**	-	0.64 ± 1.32	0.35 ± 1.00	-	0.3125	-
**Previously treated**	1.27 ± 0.59	0.20 ± 0.56	0.20 ± 0.77	**0.0002**	>0.9999	**0.0083**
**Women**	1.36 ± 0.50	0.33 ± 0.92	0.08 ± 0.28	**0.0020**	0.2500	**0.0010**
**Men**	1.00 ± 0.82	0.75 ± 1.39	0.88 ± 1.64	0.2500	>0.9999	0.7500
**Percentage of relapse-free patients¶**						
**All**	46.88	90.63	93.75	OR= 0.09 (95% IC: 0.02-0.35) **p=0.0002**	OR= 0.64 (95% IC: 0.11-3.37) p=0.6414	OR= 0.06 (95% IC:0.01-0.029) **p<0.0001**
**Responder**	54.55	100	100	OR= 0.00 (95% IC: 0.00-0.25) **p=0.0003**	-	OR= 0.00 (95% IC: 0.00-0.25) **p=0.0003**
**Non-responder**	30	70	70	OR= 0.18 (95% IC: 0.04-1.48) p=0.0736	OR= 1.00 (95% IC: 0.18-5.519) p>9999	OR= 0.18 (95% IC: 0.04-1.48) p=0.0736
**Naïve**	11.76	88.24	94.12	OR= 0.02 (95% IC: 0.00-0.14) **p<0.0001**	OR= 0.47 (95% IC: 0.03-4.45) p=0.5454	OR= 0.01 (95% IC: 0.00-0.10) **p<0.0001**
**Previously treated**	86.67	93,33	93.33	OR= 0.46 (95% IC: 0.03-4.47) p=0.5428	OR= 1.00 (95% IC: 0.05-20.23) p>0.9999	OR= 0.46 (95% IC: 0.03-4.47) p=0.5428
**Women**	50	95.83	95.83	OR= 0.04 (95% IC: 0.00-0.33) **p=0004**	OR= 1.00 (95% IC: 0.05-19.74) p>0.9999	OR= 0.04 (95% IC: 0.00-0.33) **p=0004**
**Men**	37.5	75	87.50	OR= 0.20 (95% IC: 0.03-1.63) p=0.1306	OR= 0.43 (95% IC: 0.03-4.64) p=0.5218	OR= 0.09 (95% IC: 0.01-1.00) **p=0.0389**
**Percentage of CDP-free patients¶**						
**All**	-	96.88	87.50	-	OR= 4.43 (95% IC: 0.65-55.67) p=0.1623	-
**Responder**	-	100	100	-	-	-
**Non-responder**	-	90	70	-	OR= 3.86 (95% IC: 0.45-54.81) p=0.2636	-
**Naïve**	-	100	88.24	-	OR= ∞ (95% IC: 0.47-∞) p=0.1449	-
**Previously treated**	-	93.75	87.50	-	OR= 2.14 (95% IC: 0.22-32.86) p=0.5442	-
**Women**	-	100	83.33	-	OR= ∞ (95% IC: 1-∞) **p=0.0367**	-
**Men**	-	87.50	100	-	OR= 0.00 (95% IC: 0.00-9.00) p=0.3017	-

**Percentage of patients with GdE lesions¶**						
**All**	34.38	0	6.25	OR= ∞ (95% IC: 4.27-∞) **p=0.0003**	OR= 0.00 (95% IC: 0.00-2.14) p=0.1508	OR= 7.86 (95% IC: 1.79-37.32) **p=0.0052**
**Responder**	27.27	0	0	OR= ∞ (95% IC: 2.18-∞) **p=0084**	-	OR= ∞ (95% IC: 2.18-∞) **p=0084**
**Non-responder**	50	0	20	OR= ∞ (95% IC: 1.70-∞) **p=0.0098**	OR= 0.00 (95% IC: 0.00-2.09) p=0.1360	OR= 4.00 (95% IC: 0.66-24.38) p=0.1596
**Naïve**	47.06	0	5.88	OR= ∞ (95% IC: 2.80-∞) **p=0.0012**	OR= 0.00 (95% IC: 0.00-9.00) p=0.3101	OR= 14.22 (95% IC: 1.83-165.7) **p=0.0065**
**Previously treated**	20	0	6.67	OR= ∞ (95% IC: 0.93-∞) p=0.0679	OR= 0.00 (95% IC: 0.00-9.00) p=0.3091	OR= 3.50 (95% IC: 0.45-48.22) p=0.2827
**Women**	29.17	0	0	OR= ∞ (95% IC: 2.02-∞) **p=0.0042**	-	OR= ∞ (95% IC: 2.02-∞) **p=0.0042**
**Men**	50	0	25	OR= ∞ (95% IC: 1.00-∞) **p=0.0209**	OR= 0.00 (95% IC: 0.00-2.07) p=0.1306	OR= 3.00 (95% IC: 0.35-20.32) p=0.3017
**Percentage of patients with T2w lesions§,¶**						
**All**	93.75	21.88	12.50	OR= 53.57 (95% IC: 6.48-581.4) **p<0.0001**	OR= 1.96 (95% IC: 0.51-6.49) p=0.3202	OR= 105.00 (95% IC: 10.42-1128) **p<0.0001**
**Responder**	100	9.09	0	OR= ∞ (95% IC: 20.00-∞) **p<0.0001**	OR= ∞ (95% IC: 0.47-∞) p=0.1478	OR= ∞ (95% IC: 43.62-∞) **p<0.0001**
**Non-responder**	75	50	40	OR= 3.00 (95% IC: 0.31-45.71) p=0.3932	OR= 1.50 (95% IC: 0.30-8.53) p=0.6531	OR= 4.50 (95% IC: 0.46-66.62) p=0.2367
**Naïve**	-	29.41	17.65	-	OR= 1.94 (95% IC: 0.36-8.40) p=0.4187	-
**Previously treated**	93.33	13.33	6.67	OR= 91.00 (95% IC: 7.28-1006) **p<0.0001**	OR= 2.15 (95% IC: 0.22-33.15) p=0.54288	OR= 196.0 (95% IC: 10.22-2124) **p<0.0001**
**Women**	100	16.67	8.33	OR= ∞ (95% IC: 9.95-∞) **p<0.0001**	OR= 2.20 (95% IC: 0.46-12.34) p=0.3827	OR= ∞ (95% IC: 22.17-∞) **p<0.0001**
**Men**	75	37.50	25	OR= 5.00 (95% IC: 0.46-75.79) p=0.2207	OR= 1.80 (95% IC: 0.26-12.87) p=0.5896	OR= 9.00 (95% CI: 0.73-132.1) p=0.0977

Clinical and MRI variables were measured before starting DMF treatment (baseline) and after 12 and 24 months of therapy in MS patients. Statistical significance was assessed before treatment and after 1 and 2 years in the whole sample and in the 3 subgroups of patients. p<0.05 was considered statistically significant.

†For numerical variables, p-values were calculated using the Wilcoxon signed rank test. Values are the mean +/- SD.

‡For baseline ARR, only the previous year was considered.

§ Data not available for naïve MS patients at baseline, as most of them had a single magnetic resonance imaging (MRI).

¶ For percentages, odds ratio, confidence interval and p-values were calculated using the Chi-square test.

ARR, annualized relapse rate; EDSS, expanded disability status scale; GdE, gadolinium-enhanced T1 lesions; CDP, confirmed disease progression, NEDA-3, no evidence of disease activity 3

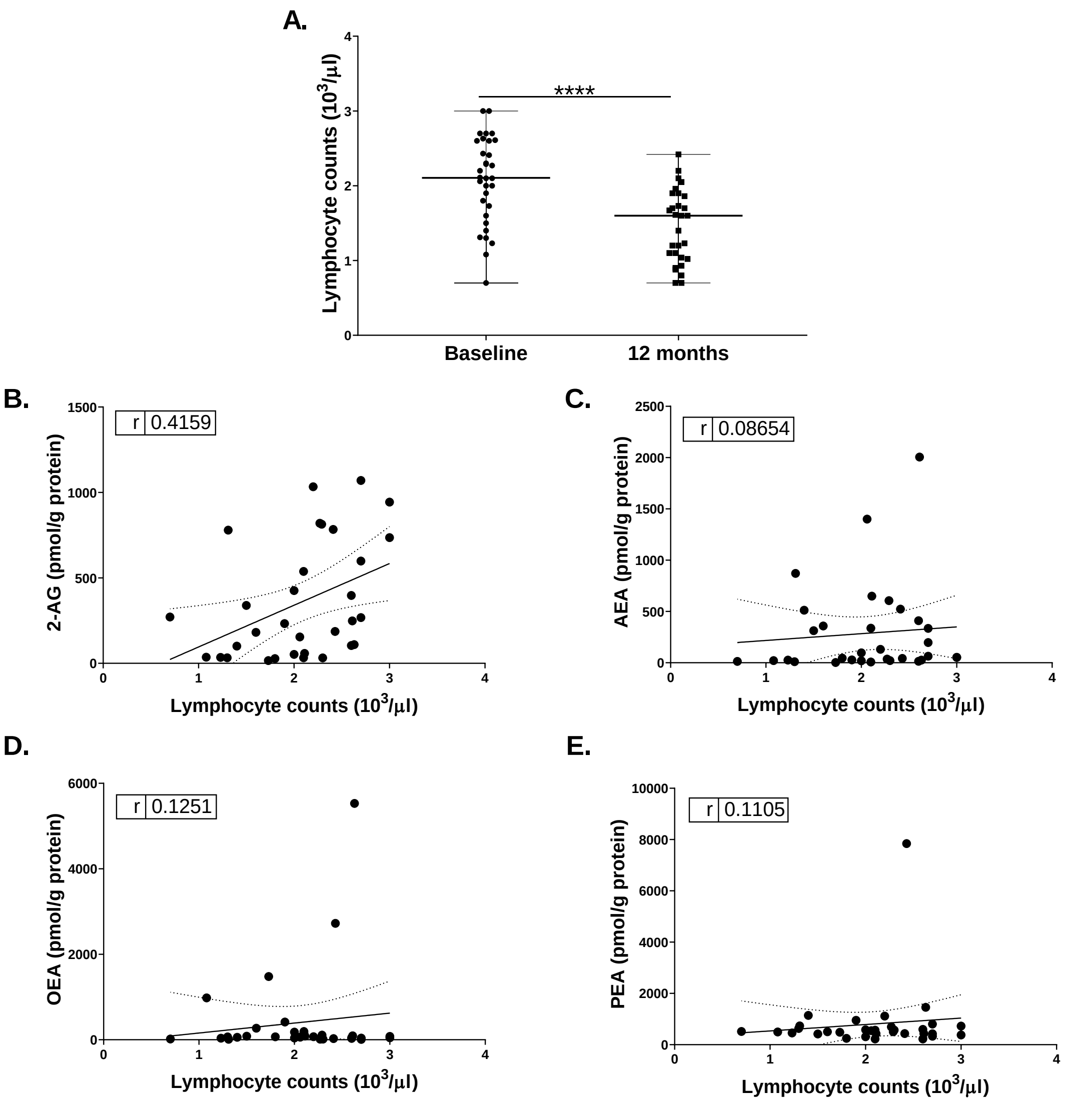

SUPPLEMENTARY FIGURE S1. Correlations between absolute lymphocyte counts and endocannabinoids at baseline. A. Lymphocyte counts at baseline and after 12 months of DMF therapy. Wilcoxon test was used to assess statistical significance. **B.** Correlation of 2-AG at baseline with lymphocyte counts at baseline. **C.** Correlation of AEA at baseline with lymphocyte counts at baseline. **D.** Correlation of OEA at baseline with lymphocyte counts at baseline. E. Correlation PEA at baseline with lymphocyte counts at baseline. B-E The Pearson correlation coefficient was calculated. ****p<0.0001

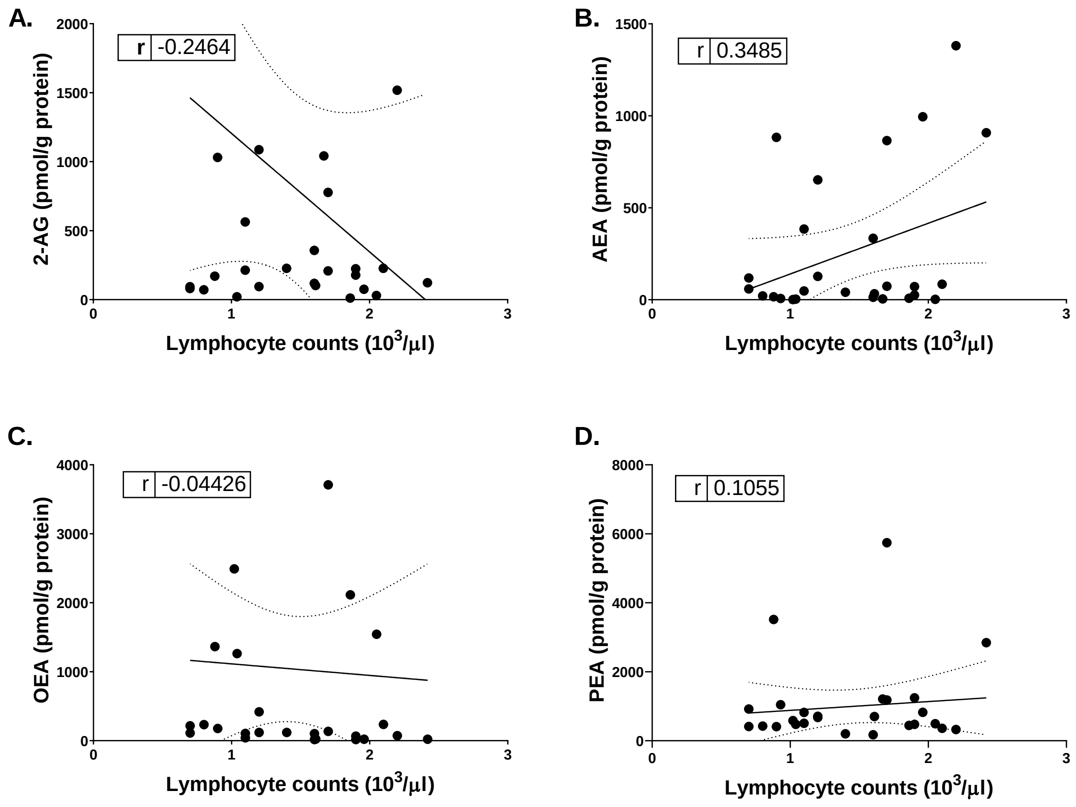

SUPPLEMENTARY FIGURE S2. Correlations between absolute lymphocyte counts and endocannabinoids at 12 months. A. Correlation of 2-AG at 12 months with lymphocyte counts at 12 months. **B.** Correlation of AEA at 12 months with lymphocyte counts at 12 months. **C.** Correlation of OEA at 12 months with lymphocyte counts at 12 months. **D.** Correlation PEA at 12 months with lymphocyte counts at 12 months. **B-E** The Pearson correlation coefficient was calculated.

A.

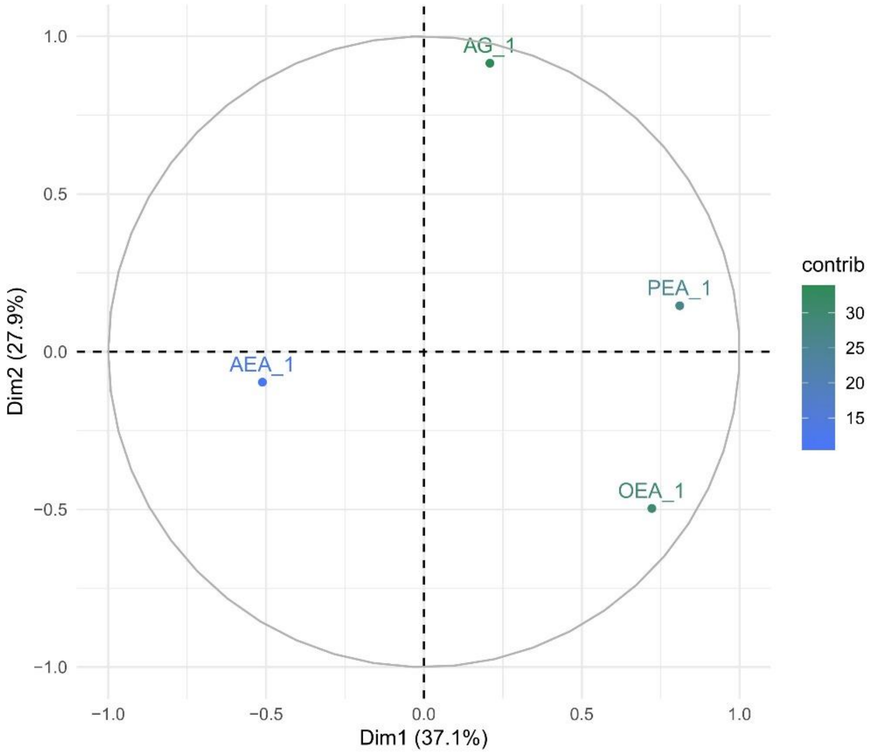

B.

	Dim. 1	Dim. 2
2-AG	2,941636	75,096820
AEA	17,653538	0,838203
OEA	35,138957	22,155966
PEA	44,265868	1,909011

SUPPLEMENTARY FIGURE S3. PCA variables contribution. A. Plot showing the relationship between the four ECBs. 2-AG, OEA and PEA are the variables more distant from the origin, meaning a high contribution to the PCA. 2-AG and PEA are positively correlated variables as they are grouped together in the same quadrant. Negatively correlated ECBs are positioned on opposite sides of the plot origin (opposed quadrants). **B.** Table showing the contribution of each ECB to the dimension 1 and 2 of the PCA. Variables that are correlated with Dim.1 and Dim.2 are the most important in explaining the variability in the data set.

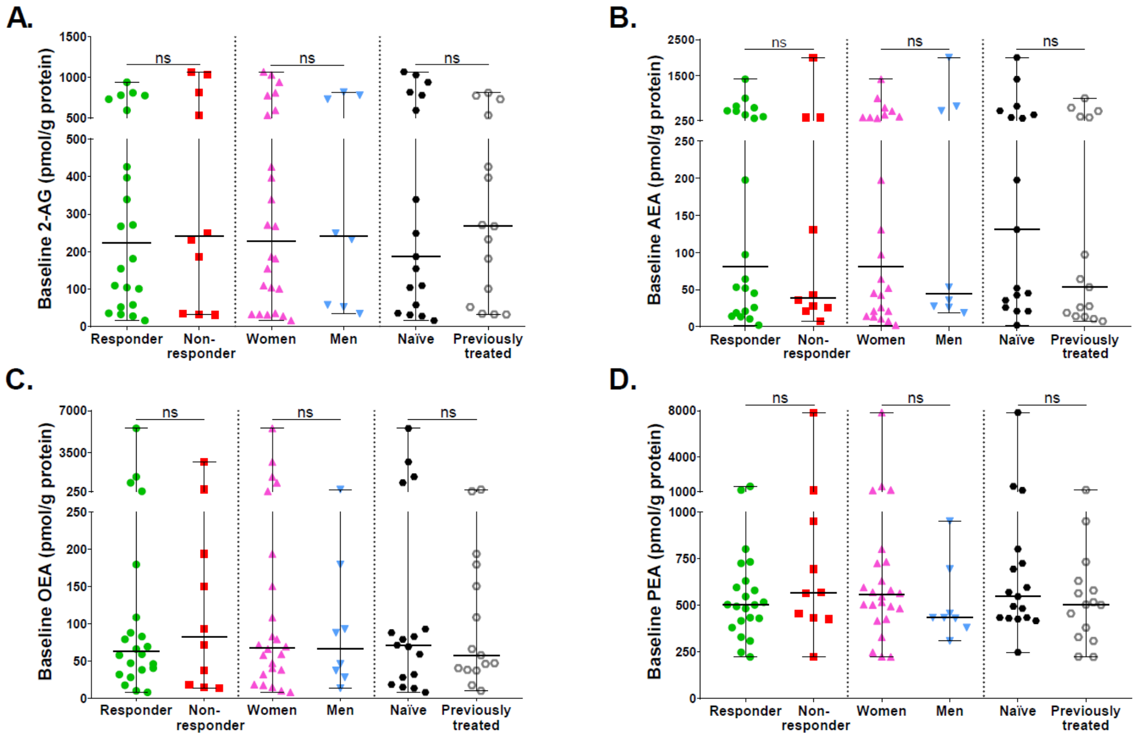

SUPPLEMENTARY FIGURE S4. Endocannabinoid levels at baseline in the subgroups of MS patients. (A-D) 2-AG, AEA, OEA and PEA baseline levels (in pmol/g of protein) in responders vs non-responders, women vs men and naïve vs previously treated patients. No differences were found for any of the ECBs in any of the subgroups. The Mann-Whitney test was used to compare differences between subgroups. Data are expressed as median with range. ns, not significant
